# Left Ventricular Assist Device Evaluation for Congestive Heart Failure With Hypereosinophilic Syndrome in a 76-Year-Old Male

**DOI:** 10.7759/cureus.30394

**Published:** 2022-10-17

**Authors:** Arooj Naz, Muhammad Hassan, Kimberley Ho

**Affiliations:** 1 Medicine, Frontier Medical and Dental College, Abbottabad, PAK; 2 Internal Medicine, Akhtar Saeed Medical and Dental College, Lahore, PAK; 3 Medicine, Ben-Gurion University, Beer Sheva, ISR

**Keywords:** left ventricular assist device (lvad), cardiac mri, loeffler endocarditis, congestive heart failure, loeffler’s syndrome, hypereosinophilic syndrome

## Abstract

Hypereosinophilic syndrome, a rare condition, is characterized by eosinophilia with associated organ infiltration and organ failure. The most commonly involved sites include the cardiovascular, gastrointestinal, and central nervous systems. Infiltration of eosinophils into the myocytes may result in congestive heart failure warranting the use of a left ventricular assist device (LVAD). The authors describe a case of a 76-year-old male with dyspnea and a consistently elevated eosinophil count. Initially diagnosed with adult-onset asthma, he failed to improve despite his adherence to recommended pharmacological treatment. The exacerbation of dyspnea combined with the signs of congestive heart failure led to the evaluation of LVAD therapy in this patient.

## Introduction

Hypereosinophilic syndrome (HES) is an uncommon hematologic disorder defined by eosinophil levels greater than 1,500/mm^3^ for more than six months without a known secondary cause [1]. Clinical manifestations of HES are highly variable and depend on the organs infiltrated by the eosinophils. They may range from asymptomatic eosinophilia to severe tissue damage and end-organ failure [2]. Infiltration of the myocardium by eosinophils is referred to as Loeffler endocarditis and may lead to congestive heart failure. Typically, Loeffler endocarditis is managed by controlling the underlying eosinophilia with corticosteroids. In addition, symptomatic treatment is required, including diuretics to reduce fluid overload and medications for heart failure therapy, including beta-adrenergic blockers, angiotensin-converting enzyme (ACE) inhibitors or angiotensin II receptor blockers (ARBs), and aldosterone antagonists. Surgical intervention may be required in patients unresponsive to medications, as evident in the clinical case of this 76-year-old male evaluated for a left ventricular assist device (LVAD).

## Case presentation

**Figure 1:**
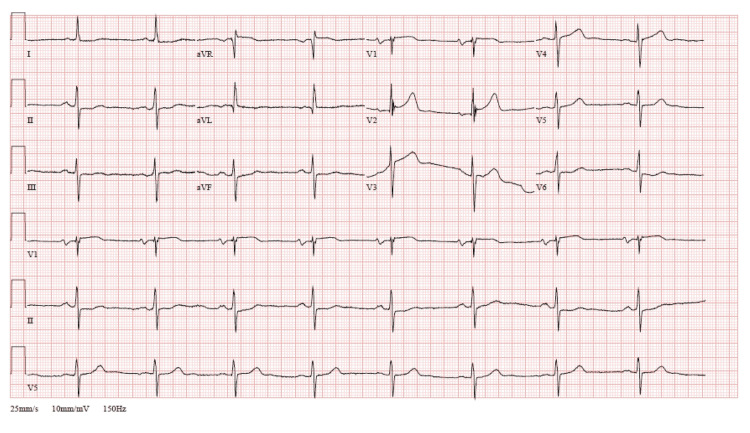
ECG

​​A 76-year-old male of Scandinavian descent presented to the emergency department (ED) with worsening dyspnea, a condition he had frequented the ED since 2019. His medical history was significant for hypertension, ​​myocardial infarction, chronic kidney disease (CKD), asthma, eosinophilic pneumonia, hypereosinophilic syndrome (HES) complicated by Loeffler endocarditis, and congestive heart failure (CHF) categorized as New York Heart Association (NYHA) class IV. He had a six-pack-year smoking history but had stopped smoking 35 years ago. He had experienced significant exertional dyspnea while exercising for the past five days, with minimal improvement after using his Symbicort (budesonide-formoterol) inhaler. His dyspnea was not present at rest, and other respiratory symptoms, including coughing and wheezing, were absent. On physical examination, systemic findings were normal except for accessory muscle use and bilateral pedal edema, and his oxygen saturation ranged between 85% and 90%. ECG, as seen in Figure 1, revealed sinus bradycardia, possible left atrial enlargement, and nonspecific ST- and T-wave abnormalities.

Echocardiography revealed an ejection fraction (EF) of 20% to 25%, a substantial reduction from the previously measured EF of 45% to 50% two years ago. His blood work revealed a continuously elevated eosinophil count despite adherence to prescribed corticosteroids, as presented in Table 1. His paracentesis sample yielded a 60% eosinophil count, and a bone marrow biopsy excluded eosinophilic leukemia as a possible diagnosis. These findings eventually led to a diagnosis of HES in 2022, and further workup was prompted to determine its effects on his underlying cardiac condition.

**Table 1:** Relevant Lab Investigations

Date of Blood Work	Leukocyte Level (Normal Count: 5 to 10 k/µL)	Neutrophils Level (Normal Count: 40%-60%)	Lymphocytes Level (Normal Count: 20%-40%)	Eosinophils Level (Normal Count: 1%-4%)	BNP (Normal Count: Less Than 100 pg/mL)
November 4, 2019	17.43 k/µL	18.6%	11.5%	64.3%	775 pg/mL
November 5, 2019	20.35 k/µL	19.3%	12.1%	63.8%	-
November 19, 2019	5.78 k/µL	81.7%	15.4%	0.3%	-
March 16, 2020	8.77 k/µL	44.0%	36.5%	12.0%	-
November 11, 2020	22.09 k/µL	21.7%	10.8%	62.2%	473 pg/mL
January 30, 2021	11.38 k/µL	77.9%	14.3%	4.1%	574 pg/mL
July 10, 2021	7.36 k/µL	53.7%	21.2%	12.6%	-
January 27, 2022	6.93 k/µL	71.4%	19.0%	3.9%	764 pg/mL
July 11, 2022	11.27 k/µL	52%	9.0%	38.0%	574 pg/mL
August 15, 2022	9.88 k/µL	91.6%	4.3%	0.0%	-
August 24, 2022	9.42 k/µL	74.8%	15.8%	0.3%	-
August 29, 2022	11.38 k/µL	94.1%	1.9%	0.0%	-
September 1, 2022	6.54 k/µL	84.2%	11.8%	0.0%	-

A cardiac biopsy of the lateral left ventricle wall was scheduled in light of an evident infiltrative pattern seen on a cardiac positron emission tomography (PET) scan. The cardiac biopsy sample revealed replacement fibrosis and myocyte hypertrophy but absent granulomas, amyloid deposition, or excess eosinophil deposition. The patient's low EF and unresponsiveness to medication prompted a discussion for left ventricular assist device (LVAD) therapy. Due to his continued fatigue and hypotension, the patient was not an optimal surgical candidate. The patient continued treatment with inotropes and cardiac rehabilitation as he awaited reevaluation for surgical intervention. As medication use continued and dosages were optimized, the patient began to experience symptomatic improvement. Investigations were repeated, and echocardiography showed an improved EF of 35% to 40%, consistent with his symptomatic improvement. Although the patient initially met the criteria for LVAD therapy, his clinical and symptomatic improvement, increased EF, and insignificant eosinophilic myocardial invasion resulted in a deferral of LVAD therapy. The patient was weaned off milrinone and deemed fit to return home with continued cardiac rehabilitation and routine follow-up.

## Discussion

**Figure 2:**
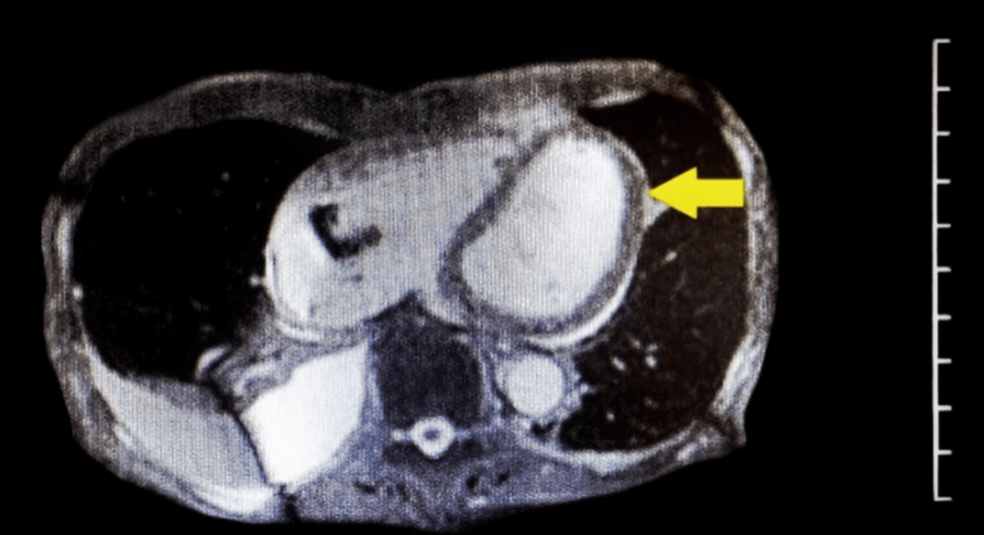
Cardiac MRI axial view showing endocarditis (arrow)

**Figure 3:**
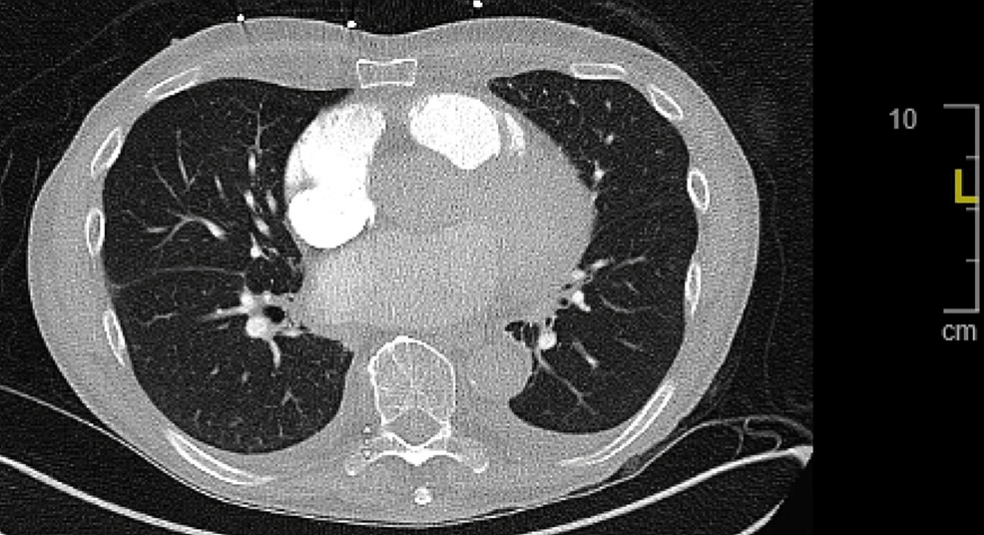
Pulmonary CT axial view showing intralobular septal thickening

Hypereosinophilic syndrome (HES) affects between 0.04 and 0.17 per 100,000 individuals [3]. This syndrome encompasses a rare and unique group of infiltrative disorders characterized by inflammation, thrombosis, and eventual fibrosis of the heart and its valves. In HES, persistent overproduction of eosinophils may infiltrate and damage multiple organs. Ultimately, these changes lead to heart failure [4], a condition defined as Loeffler endocarditis [5]. As eosinophils infiltrate the heart, they secrete protein granules that damage the endocardium and the myocardium, producing direct toxins which cause platelet activation. Activated platelets combine to form intracavitary and intravascular thrombi leading to further endocardial damage [6]. As seen in Figure 2, these pathological changes lead to fibrotic thickening of the cardiac tissue and stenosis of the heart valves, which can contribute to insufficient contraction and formation of mural thrombi. The patient's MRI yielded a left ventricular ejection fraction (LVEF) of 27%, routinely measured to be 59%-77%, and a right ventricular ejection fraction (RVEF) of 40%, typically 57%-83%. Due to the patients underlying heart failure, there were concerns for mild pulmonary edema and intralobular septal thickening, as seen in Figure 3. Loeffler endocarditis presents with signs of acute heart failure, as seen in our patient, and can be treated with medical or surgical treatment, such as a left ventricular assist device (LVAD). Pharmacological treatment includes medications typically used for heart failure, including beta-adrenergic blockers, angiotensin-converting enzyme (ACE) inhibitors or angiotensin II receptor blockers (ARBs), aldosterone antagonists, and diuretics.

LVAD is an external device that assists the heart in pumping adequate amounts of blood when the ejection fraction is <40%. It only assists cardiac activity and does not replace the heart's functionality and is therefore used as a bridge to transplant. In patients with heart failure who are unsuitable for a heart transplant, LVAD is used as destination therapy [7]. Patients with significant bleeding, infections, acute kidney injury, or brain damage are unsuitable candidates for LVAD therapy. Our patient's pulmonary capillary wedge pressure (PCWP) was approximately 15 mm Hg, and, according to Table 2, he had a moderate indication for LVAD implementation.

**Table 2:** Criteria for left ventricular assist device (LVAD) therapy Lund et al. [8].

	Strong Indication	Moderate Indication
New York Heart Association (NYHA)	NYHA IV for 60-90 days	NYHA IV for 30 days
Inotrope dependence	Chronic dependence	Intermittent dependence
Left ventricular ejection fraction (LVEF)	Less than 25%	Less than 25%
Pulmonary capillary wedge pressure (PCWP)	More than 20 mm Hg	-
Systolic blood pressure	Less than 80-90 mm Hg	-

Various imaging modalities may be used to diagnose and follow the treatment response for this rare condition. Echocardiography is the initial imaging of choice when HES is suspected. It may reveal endocardial thickening, left ventricular hypertrophy, and dilation of the affected chambers. Cardiac magnetic resonance imaging (MRI) may aid in the anatomical and functional evaluation of the cardiac chambers by detailing the presence and extension of myocardial edema and fibrosis. If noninvasive imaging is inconclusive, but the suspicion of HES is high, an endomyocardial biopsy should be conducted [9], as was done in our patient, in whom the findings were insignificant.

## Conclusions

This case report illustrated the parameters used to determine the need for a left ventricular assist device (LVAD) in the setting of hypereosinophilic syndrome (HES). In this patient, the syndrome was presumed to have led to the complication of congestive heart failure with continued deterioration. Detailed cardiac imaging and biopsies revealed unsubstantial eosinophil infiltration to initiate LVAD therapy. Instead, cardiac rehabilitation and pharmacological treatment were employed, which gradually increased his ejection fraction (EF) and allowed for the continuation of management from home with ongoing follow-up. Due to continued technological advancements, LVAD therapy, a treatment modality utilized to improve quality of life, can now be utilized in congestive heart failure due to various underlying etiologies where conventional treatment options may prove ineffective.
